# Folate Decorated Dual Drug Loaded Nanoparticle: Role of Curcumin in Enhancing Therapeutic Potential of Nutlin-3a by Reversing Multidrug Resistance

**DOI:** 10.1371/journal.pone.0032920

**Published:** 2012-03-21

**Authors:** Manasi Das, Sanjeeb K. Sahoo

**Affiliations:** Institute of Life Sciences, Bhubaneswar, India; Aristotle University of Thessaloniki, Greece

## Abstract

Retinoblastoma is the most common intraocular tumor in children. Malfunctioning of many signaling pathways regulating cell survival or apoptosis, make the disease more vulnerable. Notably, resistance to chemotherapy mediated by MRP-1, lung-resistance protein (LRP) is the most challenging aspect to treat this disease. Presently, much attention has been given to the recently developed anticancer drug nutlin-3a because of its non-genotoxic nature and potency to activate tumor suppressor protein p53. However, being a substrate of multidrug resistance protein MRP1 and Pgp its application has become limited. Currently, research has step towards reversing Multi drug resistance (MDR) by using curcumin, however its clinical relevance is restricted by plasma instability and poor bioavailability. In the present investigation we tried to encapsulate nutlin-3a and curcumin in PLGA nanoparticle (NPs) surface functionalized with folate to enhance therapeutic potential of nutlin-3a by modulating MDR. We document that curcumin can inhibit the expression of MRP-1 and LRP gene/protein in a concentration dependent manner in Y79 cells. *In vitro* cellular cytotoxicity, cell cycle analysis and apoptosis studies were done to compare the effectiveness of native drugs (single or combined) and single or dual drug loaded nanoparticles (unconjugated/folate conjugated). The result demonstrated an augmented therapeutic efficacy of targeted dual drug loaded NPs (Fol-Nut-Cur-NPs) over other formulation. Enhanced expression or down regulation of proapoptotic/antiapoptotic proteins respectively and down-regulation of bcl2 and NFκB gene/protein by Fol-Nut-Cur-NPs substantiate the above findings. This is the first investigation exploring the role of curcumin as MDR modulator to enhance the therapeutic potentiality of nutlin-3a, which may opens new direction for targeting cancer with multidrug resistance phenotype.

## Introduction

Retinoblastoma is the third most common form of cancer in infants and is an ocular disease that requires attention, as in approximately 90% of cases metastatic retinoblastoma is lethal [1]. Chemotherapy is the treatment of choice following enucleation in patients with nerve and choroid invasion and orbital extension [2]. However, their clinical use is limited by systemic toxicity, rapid blood clearance and nonspecific side effects. Further, multidrug resistance also plays a vital role in limiting the therapeutic potential of many anti-neoplastic agents in retinoblastoma. For the majority of anticancer drugs, apoptosis appears to be initiated by intrinsic or extrinsic pathways. Interestingly, an altered apoptosis regulatory pathway plays an imperative function in exhibiting chemo-resistance in retinoblastoma. Over expression of antiapoptotic protein bcl2 has been observed in retinoblastoma leading to decreased chemo-sensitivity [3]. Interestingly, retinoblastoma is caused by mutation in both alleles of the retinoblastoma gene RB1. Although, the tumor suppressor protein p53 remains functional but its activity is highly regulated by its negative regulator murin double minute (MDM2) [4]. Above all these resistance mechanisms, classical multidrug resistance (MDR) mediated via trans-membrane transporters like MRP-1 and LRP contribute to foremost resistance mechanism against various anticancer drugs in retinoblastoma. Multidrug resistance protein (MRP-1) encoded by MRP-1 gene belongs to the family of ABC membrane transporters (ABCC1) and in a similar manner as P-gp, mediates resistance to a range of structurally and functionally unrelated agents [5]. Similarly, LRP has been identified as major vault protein in human and is over expressed in 58% of retinoblastoma tumors [2]. The protein is encoded by LRP gene and mechanism of action of this protein in eliciting MDR is yet to be explored. It has been investigated intensively that, these two proteins (MRP-1 and LRP) are solely associated with multidrug resistance in retinoblastoma [6], [7]. Thus, considering the relative importance of MDR in retinoblastoma, there is an urgent call for effective therapeutic strategy in retinoblastoma therapy.

Recently the anticancer drug nutlin-3a has shown its therapeutic efficacy in diverse cancer like osteosarcoma, leukemia, colon cancer etc. [8], [9] and much attention has been given for its use in retinoblastoma therapy, because of its nongenotoxic nature [4]. Nutlin-3a is an antagonist of murin double minute (MDM2) and actively inhibits p53-MDM2 interaction. Binding to the same site on MDM2 as p53, nutlin-3a effectively inhibit MDM2-mediated p53 degradation by interfering with the molecular interaction between p53-MDM2 and results in p53 accumulation and activation [9]. In the present scenario though nutlin-3a is a potent candidate for cancer therapy, however its clinical application is limited by the fact that it acts as a substrate for multidrug resistance proteins MRP-1 and Pgp [10]. Hence, novel strategy is warranted to enhance the antiproliferative and apoptotic activity of nutlin-3a by modulating multidrug resistance. The polyphenolic compound curcumin extracted from the rhizome of turmeric (*Curcuma longa*) has been investigated intensively for its application as MDR modulator. Many research groups have shown that cucumin can effectively modulate the expression and function of major MDR proteins (Pgp, MRP, LRP, and BCRP) in diverse cancer cell lines [11]–[13]. Recently, Thiyagarajan *et al*. have explored that; curcumin can down regulate the expression of LRP protein and mRNA in a concentration dependent manner in Y79 retinoblastoma cell line [7]. Additionally, curcumin has potent anticancer properties as demonstrated in a plethora of human cancer cell line and animal carcinogenesis model [14]. Inhibition of the NFκB signaling pathway, activation of p53 pathway, suppression of bcl-2 proteins is also considered to be important for the pro-apoptotic activity of curcumin [14]–[16]. Although, curcumin is a potential modulator of MDR proteins and can target many signaling pathways, but its optimum potential is limited by poor aqueous solubility, short plasma half-life, poor bioavailability and non-specific delivery [17]. Presently, many group worked to enhance the solubility and pharmacokinetic property of native curcumin by encapsulating it in polymeric nanocarrier systems [18]. In this milieu, Mohanty *et al.* have demonstrated that encapsulation of curcumin in glycerol monooleate based nanoparticle (GMO NP) enhances its bioavailability and therapeutic activity to many folds compared to native curcumin [19]. Thus, by encapsulating curcumin in polymeric nanoparticles one can efficiently use it for anticancer therapy and/or modulating drug resistance.

Drug combinations have played a particularly prominent role in the treatment of cancer [15], [20]. Administration of a combination of agents hitting different targets and displaying different toxicity profiles can improve the therapeutic index either in the form of better efficacy and reduced toxicity. To reverse the resistance mechanisms and reduce side effects during chemotherapy, a promising approach is to combine a conventional chemotherapy with new strategies such as chemosensitizers with cytotoxic activity to inhibit ABC transporters and display antiproliferative activity [15], [21]. Recently, Patil *et al.* have shown that simultaneous delivery of tariquidar with paclitaxel in PLGA NPs overcomes multidrug resistance and enhances therapeutic effect of paclitaxel in drug resistance breast cancer cells [21]. Hence, we can anticipate that co-delivery of curcumin with nutlin-3a may emerge as a new strategy to overcome resistance of nutlin-3a in drug resistance cancer cells.

With this background, we hypothesize that, by encapsulating anti-cancer drug nutlin-3a and chemosensitizer curcumin in polymeric nanoparticles prepared from biocompatible and biodegradable polymers like PLGA, we can overcome the shortcomings associated with conventional drug delivery strategy. Further, by modulating MDR function through curcumin we can enhance the anticancer activity of nutlin-3a in drug resistance Y79 cells. Additionally, to increase the therapeutic efficacy of our dual drug loaded NPs; a targeted tumor-specific strategy is warranted. In this regard, folate receptor (FR) stands out as one of the most promising and extensively investigated epithelial cancer markers for drug targeting [19], [22]. Thus, solid tumors like retinoblastoma that are currently most difficult to treat by classical therapeutic modalities may be readily targeted with folate-linked therapeutics or carrier systems [23]. In the present study we have surface functionalized our dual drug loaded PLGA NPs with folate to interfere MDR of Y79 retinoblastoma cell. As our aim was to overcome drug resistance of Y79 cell by nutlin-3a-curcumin co-loaded NPs, we first investigated the effect of curcumin to down regulate expression of MDR proteins MRP-1 and LRP. Further, we investigated several biological parameters by cellular assays (measurement of cytotoxicity, loss of mitochondrial membrane potential, apoptosis) and found that dual drug loaded NPs contribute to a synergistic activation of antiproliferative and antiapoptotic activity in multidrug resistance Y79 cells. Hence our results demonstrated that, co-administration of nutlin-3a with an MDR modulator like curcumin, in polymeric NPs surface functionalized with folate would be significantly beneficial by augmenting the therapeutic effects and improving clinical outcomes, in cancer with multidrug resistance phenotype.

## Materials and Methods

### Reagents and cell lines

Nutlin-3a was purchased from Cayman Chemical Company (Michigan, U. S. A.) Poly (D, L-lactide-co-glycolide) PLGA, copolymer ratio 50∶50, inherent viscosity (I.V = 0.41) was purchased from Birmingham polymers, Inc. (Birmingham, AL, U. S. A.). N-hydroxysulfosuccinamide (Sulfo-NHS), 1, 3, Dicyclohexyl carbodiimide (DCC), polyvinyl alcohol (PVA, average MW 30,000–70,000), 3-[4,5-dimethylthiazol-2-yl]-2,5-diphenyltetrazolium bromide (MTT), Annexin-V-FITC, protease inhibitor cocktail, propidium iodide, 5,5′,6,6′-tetrachloro-1,1′,3,3′-tetraethylbenzimidazolocarbocyanine iodide (JC-1) and sodium dodecyl sulfate, folic acid, curcumin were purchased from Sigma-Aldrich Co. (St Louis, MO, U. S. A.). 1-ethyl-3-(3-dimethylaminopropyl) carbodiimide hydrochloride (EDC), potassium dihydrogen phosphate and Tris base were obtained from Qualigens, Mumbai, INDIA. All primary antibodies, horseradish peroxidase-conjugated secondary antibodies were purchased from Santa Cruz Biotechnology (Santa Cruz, CA, U. S. A). Y79, A549, HEK293 and SIHA cells were purchased from American Type Culture Collection (ATCC, Manassas, VA) and were grown in RPMI 1640 (Invitrogen, CA, U. S. A.) media supplemented with 10% fetal bovine serum, 100 µg/ml penicillin G and 100 µg/ml streptomycin (Invitrogen, USA) at 37°C in a humidified atmosphere containing 5% CO_2_ (Hera Cell, Thermo Scientific, Waltham, MA).

### Preparation of drug loaded PLGA nanoparticles

Drug loaded PLGA NPs were formulated by oil-in-water single emulsion-solvent evaporation technique [24]. In brief, a solution of 100 mg PLGA polymer and 10 mg drug (nutlin-3a or curcumin) corresponding to 10% w/w dry weight of polymer in 3 ml of chloroform was emulsified in 12 ml of 2% w/v aqueous solution of PVA to form an oil-in-water emulsion. In case of dual drug loaded nanoparticles, both the drugs were added in 1∶1 ratio such that the total drug in the formulation corresponded to 10% w/w dry weight of polymer. The emulsification was carried out using a micro-tip probe sonicator set at 55 W of energy output (VC 505, Vibracell Sonics, Newton, USA) for 2 min over an ice bath. The emulsion was stirred overnight at room temperature to allow evaporation of organic solvent and formation of NPs. NPs were recovered by ultracentrifugation at 1,00,000 g for 20 min at 4°C (Sorvall Ultraspeed Centrifuge, Kendro, USA), washed twice with water to remove excess PVA and unencapsulated drug. The nanoparticulate suspension was then lyophilized for 2 days (−47°C and <10 µm mercury pressure, LYPHLOCK 12, Labconco, Kansas City, MO) to obtain the powdered NPs for further use.

### Surface functionalization of drug loaded NPs with folate ligand

In brief, folic acid was reacted with DCC and NHS in DMSO at stoichiometric molar ratio of Fol/DCC/NHS = 1/1.2/2 for 6 hrs at 50^°^C [25]. The product was filtered to remove N, N-dicyclohexylurea (DCU). The activated folate was then reacted over night with ethylene diamine in presence of pyridine as catalyst. The aminated folate (Fol-NH_2_) was precipited out by adding excess cold acetonitrile, followed by vacuum filtration. For conjugation of Fol-NH_2_ onto the surface of PLGA NPs, 10 mg of drug loaded NPs (nutlin-3a loaded NPs (Nut-NPs), curcumin loaded NPs (Cur-NPs), nutlin-3a and curcumin loaded NPs (Nut-Cur-NPs)) were dispersed in 5 ml of PBS (0.02 M, pH 7.4) followed by addition of 250 µl of EDC (1 mg/ml) and 250 µl of NHS (1 mg/ml) to the above suspension. EDC activation was carried out by agitating the above suspension for 2 hrs at room temperature using a magnetic stirrer. Excess of unreacted EDC and NHS were removed and activated NPs were recovered by ultracentrifugation at 1, 00, 000 g for 20 min at 4°C (Sorvall Ultraspeed Centrifuge, Kendro, USA). The activated NPs were dispersed in 2 ml of PBS (0.02 M, pH 7.4) followed by addition of 100 µl of aminated folate solution (1mg/ml in PBS). The solution was again agitated for 2 hrs at room temperature and excess of unconjugated folate was removed by ultracentrifugation. The recovered folate conjugated NP suspension was lyophilized for further use [26]. Conjugation of folate onto PLGA NPs surface was confirmed by FTIR analysis (Methods S1).

### Characterization of drug loaded PLGA NPs

#### Particle size analysis and zeta potential measurement

Particle size and size distribution (polydispersity index) of the formulations were determined, by dynamic light scattering (DLS), using a Zetasizer (Nano ZS, ZEN3600, Malvern Instrument, UK) with a wavelength of 532 nm at 25^o^C with an angle detection of 90^o^ [27]. In brief, ∼ 1 mg/ml of nanoparticulate suspension were prepared in MilliQ water, sonicated for 30 sec over an ice bath using a sonicator (VC 505, Vibracell Sonics, Newton, USA) set at 55 W of energy output. A 100 µl of the above NPs suspension was diluted to 1 ml in water and then subjected to particle size measurement. Zeta potential of the nanoformulations was determined by the same instrument, following the above protocol. All measurements were performed in triplicates.

#### Transmission electron microscopy (TEM)

Nanoparticles were also evaluated for size by transmission electron microscope (Philips/FEI Inc. Barcliff, Manor, NY). For this purpose, a sample of NPs (0.5 mg/ml) was suspended in water and sonicated for 30 sec. One drop of this suspension was placed over a carbon coated copper TEM grid (150 mesh, Ted PELLA Inc. Rodding, CA) and negatively stained with 1% uranyl acetate for 10 min, allowed to dry and the images were visualized at 120 kV under microscope.

#### Evaluation of encapsulation efficiency of nutlin-3a and curcumin

Encapsulation efficiency was determined by the reverse phase isocratic mode of high performance liquid chromatography (RP-HPLC) method, using Agilent 1100 HPLC (Agilent technologies, Waldbronn Analytical Division, Germany) which consists of Zorbax Eclipse XDB-C18, 150×4.6 mm, i.d with internal standard of dimethylphthalate. To estimate entrapped drugs in NPs, 5 mg of freeze dried drug loaded NPs (containing single drug or dual drug) was dissolved in 5 ml of acetonitrile and kept in the shaker at 37°C and 2 g (Wadegati Labequip, India) for 2 days. The samples were then centrifuged at 17,458 g for 10 min at 25°C (SIGMA 3K30, Munich, Germany) to extract the drug present in the solution. The collected supernatant was analyzed for drug content by RP-HPLC using specific mobile phase for each drugs, at a flow rate of 1 ml/min, at 30°C with thermostat (Model No -G1316A) [28], [29]. The percentage encapsulation efficiency of each drug was calculated by dividing the amount of drug entrapped by the total amount of drug used in formulation, multiplied by 100.

#### *In vitro* release kinetics of different encapsulated drugs from NPs

The release kinetics of drugs from NPs was carried out in an *in vitro* condition [30]. In brief, 10 mg of drug loaded unconjugated and folate conjugated NPs was dispersed in 3 ml of PBS (0.01 M, pH 7.4) containing 0.1% v/v of Tween 80 (which was added to maintain a sink condition for hydrophobic drug). The nanoparticulate suspension was equally divided in three tubes 1 ml each (as experiment was performed in triplicates) and kept in a shaker at 37°C at 2 g (Wadegati Labequip, India). At particular time intervals samples were taken out from shaker and centrifuged at 17,458 g, 25°C (SIGMA 3K30, Munich Germany) for 10 min. The collected supernatants were lyophilized for 2 days. To the pellet obtained after centrifugation, 1 ml of fresh PBS-Tween 80 solution was added and placed in shaker for next readings. The lyophilized sample was dissolved in 1 ml of acetonitrile and kept in the shaker at 37°C and 2 g (Wadegati Labequip, India) for 2 days. After 2 days, the sample was centrifuged at 17,458 g for 10 min at 25°C to collect the drug in supernatant. 20 µl of this supernatant was injected in the HPLC to determine the amount of drug released with respect to different time intervals.

### Expression study of MDR mRNA and proteins

To detect expression of major MDR genes, Y79, A549 (MRP-1 and LRP positive), SIHA (LRP negative) and HEK (MRP-1 negative) cell lines (at a density of 50×10^5^ cells) were taken and total endogenous mRNA from all above mentioned cells was isolated using the RNeasy Mini kit (Qiagen, Hamburg, Germany) according to the manufacturer's instructions. RNA was treated with DNAse (Qiagen, Hamburg, Germany) to eliminate any DNA contamination. Total RNA (l µg) was reverse-transcribed to cDNA using M-MLV reverse transcriptase and oligo (dT) primer (Fermentas International INC, Ontario, Canada) by incubating the reaction mixture at 37°C for 60 minutes, 72°C for 5 minutes and the resulting first-strand cDNA was used as template in the semiquantitative polymerase chain reaction (PCR). The cDNA product was subjected to PCR (Vapo protect, eppendorf) for 25 cycles by LRP and MRP-1 specific primers (Table 1). The primers used to amplify cDNA by PCR were designed using Primer Express software. β-actin serve as an internal control. The PCR products were electrophoresed on ethidium bromide stained 2% agarose gel [7]. All samples were analyzed in triplicates. To investigate expression of LRP and MRP-1 proteins, cells were lysed in lysis buffer (1 M Tris, pH 7.5, 150 mM NaCl, 1% Nonidet P-40, 10% glycerol, 100 mM EDTA, protease inhibitor cocktail). Aliquots of each sample containing 100 µg proteins were resolved in SDS-polyacrylamide gel, and transferred to polyvinylidene difluoride (PVDF) membranes. Membranes were then probed with primary antibodies diluted at 1∶1000 v/v for MRP-1 and LRP. The membranes were reprobed with anti-β-actin antibody as loading control after being stripped with stripping buffer. The membrane were washed three times with PBS containing 1% Tween-20 and probed with secondary antibody. Proteins were visualized by using enhanced chemiluminescence detection reagents (Amersham Biosciences, Arlington Heights, IL) and exposed to Kodak life science imaging film (Kodak, USA) [28]. Experiment was repeated at least thrice.

**Table 1:** Primers used in PCR analysis.

	Forward primer	Reverse primer	Product size (bp)
MRP-1	ATGTCACGTGGAATACCAGC	GAAGACTGAACTCCCTTCCT	155
LRP	TGGCTTTGAGACCTCGGAAG	CCAGTCTCTGAGCCTCATGC	230
β-actin	CTGGCACCACACCTTCTACAAT	AATGTCACGCACGATTTCCCGC	380
bcl2	GGATTGTGGCCTTCTTTGAG	CCAAACTGAGCAGAGTCTTC	238
NFκB	TCGTTTCCGTTATGTATGT	CCTTGGGTCCAGCAGTTA	227

### MDR gene expression following curcumin treatment by real time RT-PCR

In brief, Y79 cells (50×10^5^ cell density) were treated with 2–12 µg/ml concentrations of native curcumin for 2 days. Following various treatments, total endogenous mRNA was isolated using the RNeasy Mini kit (Qiagen, Hamburg, Germany). RNA was treated with DNAse (Qiagen, Hamburg, Germany) to eliminate any DNA contamination. Total RNA (l µg) was reverse-transcribed to cDNA and the resulting first-strand cDNA was used as template in the real-time PCR. The primers used to amplify cDNA by real-time PCR were designed using Primer Express software (Table 1). Real-time monitoring of PCR amplification of cDNAs was done using qPCR Master Plus for SYBR Green I-DTTP (Eurogentec, Belgium) with gene specific primers, in real time PCR (DNA Engine Opticon2, MJ Research Incorporated, BioRad, Philadelphia, USA). All samples were done in triplicates. Relative quantification of gene expression was performed using β-actin as an internal control [10].

### Investigation of MDR protein expression following curcumin treatment

To explore the effect of curcumin on the expression of LRP and MRP-1, Y79 cells (10×10^5^ cell density) were treated with 2–12 µg/ml concentrations of native curcumin for 2 days. Following various treatments, protein was extracted and 100 µg proteins were subjected to western blot analysis with MRP-1 and LRP specific primary antibody. Experiment was performed in triplicates [7]. Further, the effect of curcumin loaded nanoformulation (Cur-NPs, Fol-Cur-NPs) on the expression of LRP and MRP 1 proteins in comparison to native curcumin was investigated following treatment of cells with 2 µg/ml curcumin in native form or nanoformulation for 2 days.

### Cellular uptake study by flow cytometry

Cellular uptake efficiency was studied using native curcumin, curcumin loaded NPs and folate conjugated curcumin loaded NPs (Fol-Cur-NPs) [28], [31]. As curcumin has its intrinsic fluorescence property it serve as a fluorescence probe to efficiently investigate the uptake of drug loaded NPs. Briefly, 6 well plates (Corning, NY, USA) were seeded with Y79 cells (folate receptor +ve) and A549 cells (folate receptor −ve) at 100,000 cells per well density and incubated with 1 ml of freshly prepared medium containing native curcumin, Cur-NPs, Fol-Cur-NPs (equivalent to 10 µg/ml concentrations of native curcumin) for 2 hrs at 37°C in CO_2_ incubator (Hera Cell, Thermo Scientific,Waltham, MA). Cells treated with only medium were used as respective controls. At the end of the incubation period, the cells were collected and washed three times with cold DPBS to eliminate excess of native curcumin or NPs, which were not taken up by the cells. In all FACS analysis (FACScan flow cytometer, Becton Dickinson), cell debris and free particles were excluded by setting a gate on the plot of side-scattered light (SSC) vs forward-scattered light (FSC). A total of 10,000 ungated cells were analyzed. The increase of fluorescence intensity in the cells treated with native curcumin, and curcumin loaded NPs relative to that of untreated control cells was expressed as mean fluorescence increase relative to control. To verify that the uptake is mediated via folate receptor, a competitive experiment in presence of varying concentration of free folate was performed. For the above study Y79 cells were treated with (0–30 mM) of folate for 1 hr prior to incubation with Cur-NPs, Fol-Cur-NPs or native curcumin and then subjected to above protocol.

### Qualitative cellular uptake analysis by confocal laser scanning microscopy

Y79 and A549 cells were seeded in three sets in Bioptech® tissue culture plates (Bioptechs Inc. Butler, PA) at a density of 1×10^5^ cells/ml and incubated overnight at 37°C. Cells were treated with either 10 µg/ml of native curcumin or Cur-NPs and Fol-Cur-NPs (containing equivalent concentration of curcumin) in growth medium for 2 hrs. At the end of the incubation period, the cells were rinsed with DPBS three times and fixed with 70% ethanol for 20 min at 37°C. Subsequently cells were rinsed with DPBS and 1 µg/ml PI solution was added to stain cell nucleus for 30 min. PI was washed three times using DPBS and cells were visualized by confocal laser scanning microscope (Leica TCS SP5, Leica Microsystems GmbH, Germany) equipped with an argon laser using FITC filter (Ex(λ) 488 nm, Em(λ) 525 nm) and PI filter (Ex(λ) 530 nm, Em(λ) 615 nm) [28]. Curcumin and PI showed green colour and red colour respectively.

### *In vitro* cellular cytotoxicity assay

The effect of native drugs and drug loaded nanoformulations on cell proliferation was determined by MTT based colorimetric assay [32]. Briefly, folate receptor expressing Y79 cell line and folate receptor non-expressing cell line A549 were seeded in 96-well plates (Corning, NY, USA) at density 4000 cells per well. After the overnight incubation at 37°C, cells were treated with varying concentrations of drugs (either as single native drug or encapsulated in NPs or dual drugs in solution or encapsulated in NPs). Cell viability was determined on 5^th^ day, following drug treatment using MTT assay. A 10 µl/well MTT solution (5 mg/ml) was added, plates were incubated at 37°C for 3 hrs, and then the media was replaced with 100 µl of DMSO to dissolve the formazan crystals. The absorbance was measured at 540 nm using a microplate reader (Synergy HT, BioTek® Instruments Inc. Winooski, VT, USA). The effect of each treatment was calculated as a percentage inhibition against the respective untreated controls. The IC_50_ was determined by nonlinear regression analysis using the equation for a sigmoid plot [33].

### Cell cycle analysis by flow cytometry

Cell cycle is an important phenomenon that plays a crucial role in developmental pathways, and is frequently deregulated in many cancer diseases. The distribution of DNA in cell cycle was studied by flow cytometry [28]. In brief, 1×10^5^ cells/ml were seeded in 25 cm^2^ culture flasks (Corning, NY, USA) containing 5 ml media and incubated overnight at 37°C. Next days, 5 ml of media each containing 1.5 µg/ml concentrations of nutlin-3a (in solution or nanoformulations) or 2 µg/ml of curcumin (in solution or nanoformulations) or dual drugs (1.5 µg/ml nutlin-3a+2 µg/ml curcumin) in solution or nanoformulations were added to the flasks and the cells were incubated for 24 hrs in CO_2_ incubator at 37°C. Cells treated with only medium was used as control. After incubation time period, the cells were collected by centrifugation at 91 g (SIGMA 3K30, Munich, Germany) and washed twice with DPBS. The collected cells were resuspended in 500 µl of hypotonic propidium iodide solution which consists of 1 µl of propidium iodide (1 µg/µl), 1 µl of RNase A (10 µg/µl, MP Biomedicals, Inc. Germany), and 0.5% Tween 20 in 500 µl of PBS (0.01 M, pH 7.4) and incubated for 20 min at room temperature in dark before analysis. Cell cycle distribution of the cells was determined by analyzing 10,000 ungated cells using a FACScan flow cytometer and Cell Quest software (FACS Calibur; Becton-Dickinson, San Jose, CA). All experiments were performed in triplicates.

### Measurement of loss of mitochondrial membrane potential (MMP)

Detection of the mitochondrial permeability transition event provides an early indication of the initiation of cellular apoptosis. Changes in the mitochondrial membrane potential were measured by flow cytometry using JC-1 dye (cationic dye, which exhibits potential-dependent accumulation in mitochondria) [28]. Briefly, 1×10^5^ cells/ml were seeded in 25 cm^2^ culture flasks (Corning, NY, USA) containing 5 ml media and incubated overnight at 37°C. After overnight incubation, cells were treated with 5 ml of media each containing 1.5 µg/ml concentrations of nutlin-3a (in solution or nanoformulations) or 2 µg/ml of curcumin (in solution or nanoformulations) or dual drugs (1.5 µg/ml nutlin-3a+2 µg/ml curcumin) in solution or nanoformulations and then incubated for 2 days in CO_2_ incubator at 37°C. Cells treated with only medium was used as control. After completion of incubation period, cells were collected by centrifugation at 91 g (SIGMA 3K30, Munich, Germany) and washed twice with DPBS. The collected cells were then treated with JC-1 staining solution (1 µg/ml JC-1 in DPBS warm to 37°C) and incubated at 37°C for 20 min in a CO_2_ incubator. After incubation period, cells were washed twice with DPBS. 10,000 cells were examined for each sample on a FL-1 (530 nm) *versus* FL-2 (585 nm) dot plot on a Becton Dickinson FACSort. Experiment was performed in triplicates.

### *In vitro* apoptosis studies

Apoptosis is a novel form of cell death, which plays a major role during development, normal tissue homeostasis, and is deregulated in many diseases including cancer. Induction of apoptosis in Y79 cells following drug treatment was studied by flow cytometry [28]. In brief, 5×10^5^ cells were allowed to grow in 25 cm^2^ culture flasks (Corning, NY, USA) containing 5 ml media for overnight at 37°C. Subsequently, 5 ml of media each containing 1.5 µg/ml concentrations of nutlin-3a (in solution or nanoformulations) or 2 µg/ml of curcumin (in solution or nanoformulations) or dual drugs (1.5 µg/ml nutlin-3a+2 µg/ml curcumin) in solution or nanoformulations were added to the flasks and the cells were incubated for 2 days in CO_2_ incubator at 37°C. Untreated cells were used as respective control. At predetermined time period, the cells were collected by centrifugation at 91 g (SIGMA 3K30, Munich, Germany) and washed twice with DPBS. The pelleted cells were resuspended in 500 µl of 1X binding buffer, 5 µl Annexin V-FITC (1 µg/µl) and 1 µl propidium iodide (1 µg/µl) and incubated in dark for 10 min. The apoptotic cells were determined by analyzing 10,000 gated cells using a FACScan flow cytometer and Cell Quest software. All experiments were performed in triplicates.

### Analysis of apoptotic signal proteins by western blotting

For western blot analysis, 3×10^6^ number of Y79 cells were treated with 1.5 µg/ml concentrations of nutlin-3a (in solution or nanoformulations) or 2 µg/ml of curcumin (in solution or nanoformulations) or dual drugs (1.5 µg/ml-nutlin-3a and 2 µg/ml-curcumin) in solution or nanoformulations for 2 days. Following various treatments, cells were lysed with lysis buffer and 100 µg of extracted proteins was subjected to western blotting with specific primary antibody [26]. Experiment was repeated at least thrice.

### Real time quantitative PCR analysis

To explore the effect of drug combination over single drugs in suppressing antiapoptotic signaling pathways, a quantitative expression study of NFκB and bcl2 at gene level was investigated by real time quantitative PCR analysis [10]. Briefly, 3×10^6^ number of Y79 cells were incubated with 1.5 µg/ml concentrations of nutlin-3a (in solution or nanoformulations) or 2 µg/ml of curcumin (in solution or nanoformulations) or dual drugs (1.5 µg/ml nutlin-3a and 2 µg/ml curcumin) in solution or nanoformulations for 2 days. Total mRNA was isolated and real-time PCR amplification of cDNAs was done using above protocol. Relative quantification of gene expression was performed using β-actin as an internal control.

**Figure 1:**
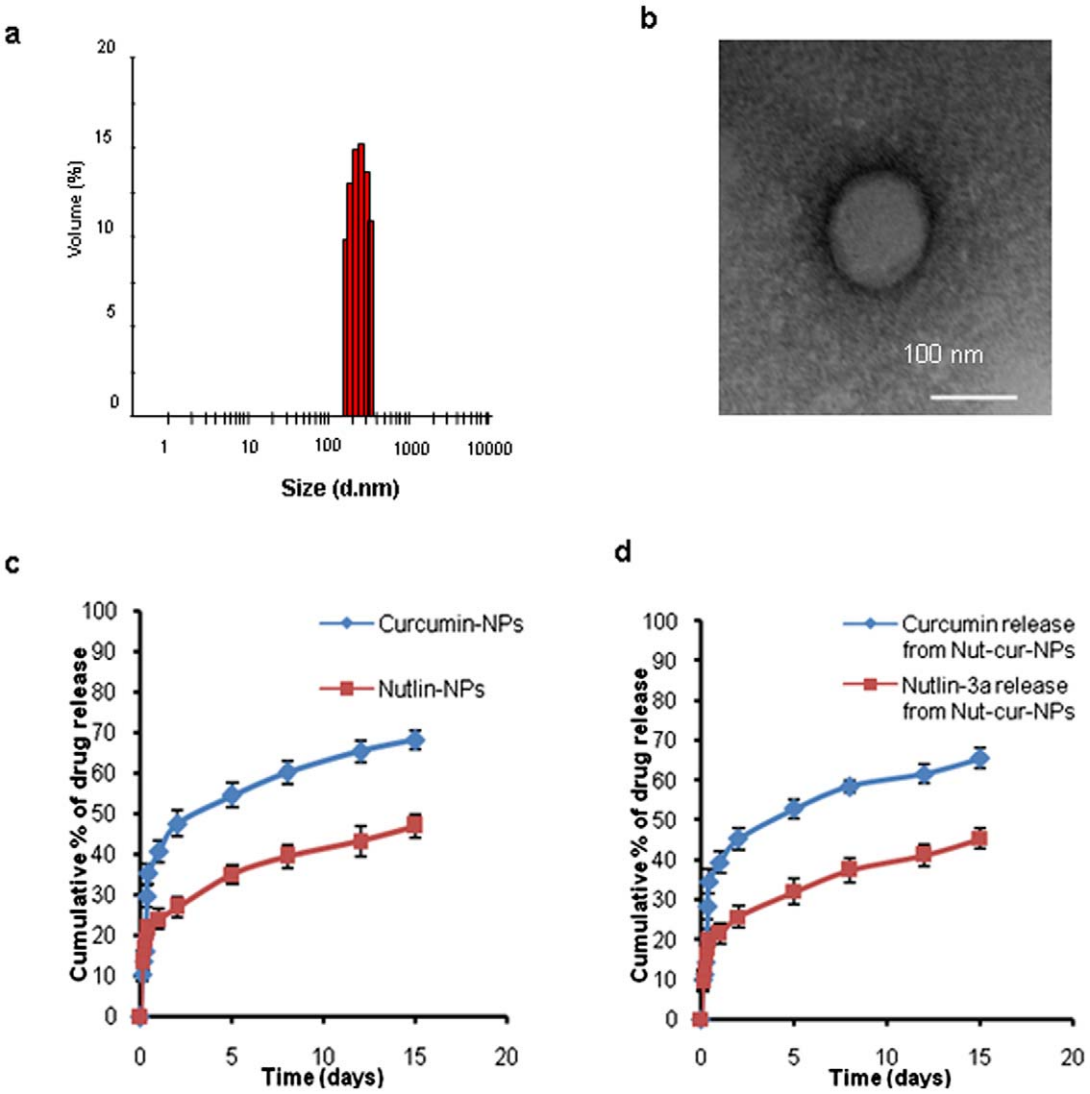
Physiochemical characterization of nanoformulations. Size of NPs (a) was measured by DLS. (b) TEM analysis of drug loaded NPs depicting the spherical nature of the nanoformulation. Scale bar is 100 nm. (c and d) Sustain release phenomenon exhibited by drug loaded nanoformulations. The values are shown as mean±SD, n = 3.

### Statistical analysis

All experiments were repeated at least three times. Statistical analyses were performed using a Student's *t* test. Values of p<0.05 (*) were indicative of significant differences and very significant difference if p<0.005 (**).

## Results

### Physiochemical characterization of drug loaded PLGA nanoparticles

**Figure 2:**
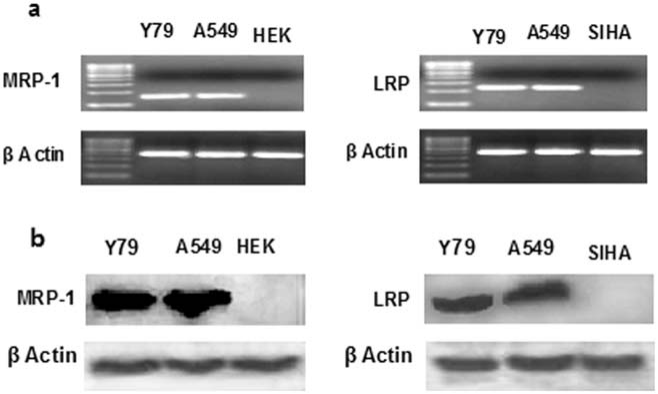
Expression study of MDR mRNA and proteins. Expression of LRP and MRP-1 mRNA (a) and proteins (b) was determined by semiquantitative PCR and western blotting respectively. RNA was extracted and PCR was performed with gene specific primers. The PCR products were run in 2% agarose gel. β-actin serves as control for equal loading. The expression of MDR proteins was further confirmed by western blot analysis (b). Cells were lysed and expression of MDR proteins was analysed by respective primary antibodies. A549 cells act as positive control for LRP and MRP-1 expression, while HEK and SIHA serve as negative control for MRP-1 and LRP expression respectively. The results are representative of three independent experiments.

In an attempt to formulate site specific drug delivery system, drug loaded PLGA nanoparticles were synthesized by single emulsion method for targeting folate receptor over expressing retinoblastoma cells. The two-step EDC/NHS activation method enables the conjugation of amino groups of aminated folate to the carboxyl groups of PLGA through an amide bond formation [25], [34]. Conjugation of folate to PLGA NPs was confirmed by FTIR analysis and the amide bond peak at 1630.11 cm^−1^ in folate functionalized NPs clearly indicates the formation of amide bond following conjugation (Results S1, Figure S1) The conjugation yield was found to be 8.5 mg and it was estimated that approximately 8 µg of folate was conjugated per mg of nanoparticles. An efficient nanoparticulate system should have a high loading capacity to minimize the quantity of carrier required for administration. In the present study we have achieved an efficient loading of both the drugs (curcumin and nutlin-3a) in PLGA NPs, when used singly or in combination (Table 2). Folate conjugated NPs showed an entrapment efficiency of drug comparable to that of unconjugated NPs. Similar result was evident from experimental work of Misra *et al.* in doxorubicin loaded NPs and NLS conjugated doxorubicin loaded NPs [30]. DLS analysis revealed that the formulated NPs had a unimodal size distribution with a mean hydrodynamic diameter of ∼ 250 nm having polydispersity index ∼ 0.1 and a negative zeta potential of ∼ −18 mV (Figure 1a, Table 2). Surface modification of PLGA-NPs with folate had no significant effect on its physical characteristics. A similar trend was also observed by Tseng *et al.* when they conjugated biotinylated epidermal growth factor (EGF) on gelatin nanoparticles [35]. TEM images (Figure 1b) showed discrete spherical outline and monodispersed size (∼ 100 nm) of PLGA NPs. The higher hydrodynamic diameter of NPs achieved by DLS analysis as compared to the size obtained by TEM analysis may be contributed by the hydration of the surface associated PVA [30]. Sustained release of entrapped drug from NPs is an important parameter for developing successful formulations, as it enables constant amount of drug persistently at site of action. As shown in Figure 2c and d, the release profile of single drugs or drugs in combination from nanoparticulate system exhibited a biphasic drug release pattern that was characterized by a initial rapid release followed by a slower continuous release phase over 15 days. The release of drugs from folate conjugated NPs followed more or less similar release pattern to that of unconjugated NPs (data not shown).

**Table 2:** Physico-chemical characterization of nanoparticle formulation.

Formulation	Size (nm)^a^	Zeta Potential (mV)^b^	Polydispersity Index	Entrapment efficiency (%)^c^
Nut-NP	226±7.3	−18.4±0.57	0.14±0.008	65±4.3
Fol-Nut-NPs	234±6.5	−16.8±0.8	0.11±0.005	63±1.4
Cur-NP	227±5.3	−17.9±0.37	0.18±0.007	86±5.8
Fol-Cur-NPs	236±5.5	−16.2±0.49	0.12±0.009	77±2.1
Nut-Cur-NP	229±6.3	−18.9±0.87	0.14±0.005	64±3.2 (nutlin), 85±3.3 (curcumin)
Fol-Nut-Cur-NPs	235±4.6	−15.9±0.59	0.16±0.008	63±1.2 (nutlin) 84±2.4 (curcumin)

a Size in nm was measured by Zetasizer.

b Zeta potential in mV was measured by Zetasizer.

c Polydispersity Index was measured by Zetasizer.

Entrapment efficiency of different drugs were measured by HPLC.

Size was measured by DLS using a Zetasizer. The surface charge was also measured by Zetasizer.

### Expression of LRP and MRP-1 at m RNA and protein level in Y79 cell*s*

LRP and MRP-1 are the major MDR proteins conferring drug resistance in retinoblastoma [6], [7]. Presently, we have assessed the expression of MRP-1 and LRP at mRNA and protein level in Y79 cells by semiquantitative PCR and western blotting technique respectively. In the current study, we have found that the model retinoblastoma cell line Y79 expresses significant level of LRP and MRP-1 mRNA and protein as evident from Figure 2a and b.

### Investigation of LRP and MRP-1 mRNA and protein expression following curcumin treatments

**Figure 3:**
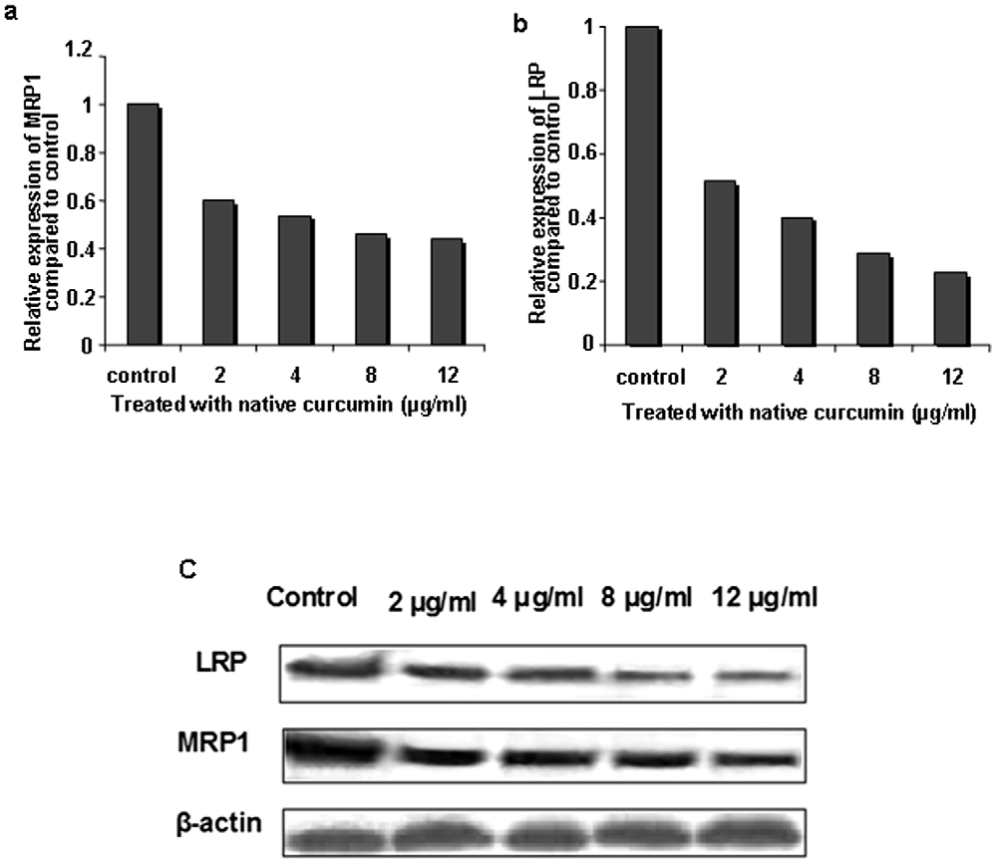
qRT PCR analysis of the effect of curcumin treatment on MRP-1 and LRP gene/protein expression. Y79 c ells were treated with increasing concentration of native curcumin for 48 hrs and relative expression of MRP-1 and LRP mRNA was measured (a and b). β-actin serves as internal control. Dose dependent inhibition of MDR mRNA was evident with curcumin treatment (a and b). Dose dependent inhibition of MRP-1 and LRP protein expression following curcumin treatment was also confirmed by western blotting (c).

Intense investigations have been performed by several research groups, proving the potential of curcumin as MDR modulator [11]–[13], [30], but very few have been reported depicting the role of curcumin in down regulating MDR gene expression. Here, we have treated Y79 cells with increasing concentration of native curcumin (2–12 µg/ml) for 2 days and examine the mRNA expression by quantitative RT-PCR. The result clearly indicates a concentration dependent down regulation in expression of MRP-1 and LRP mRNA following curcumin treatment (Figure 3a and b). It is noteworthy that, cells treated with lowest concentration of curcumin (2 µg/ml) revealed fifty fold decreases in expression level of LRP mRNA. A similar trend was also observed for MRP-1 mRNA expression. The above results evidently indicates that, curcumin plays significant role in regulating MDR at transcriptional level; so, we have further assessed its effect at protein level by western blotting. Interestingly, reduced expression of LRP and MRP-1 protein was found, following treatment with increase concentration of native curcumin (Figure 3c). The results clearly showed a significant decrease in expression of LRP and MRP-1 proteins in cells treated with 2 µg/ml curcumin. As, the result indicates a significant inhibition in expression of LRP and MRP-1 at gene and protein level with 2 µg/ml of native curcumin treatment, we further investigated the effect of nanoformulation in modulating the expression of these MDR proteins in comparison to native curcumin at this particular concentration (Results S1, Figure S2). The result evidently indicate that, folate targeted curcumin loaded NPs is able to inhibit the expression of LRP and mRP-1 proteins in an enhanced way compared to unconjugated counterpart and native curcumin, pointing towards the therapeutic potential of targeted NPs in overcoming multidrug resistance (Figure S2).

### Assessment of cellular uptake of different curcumin loaded nanoformulations

**Figure 4:**
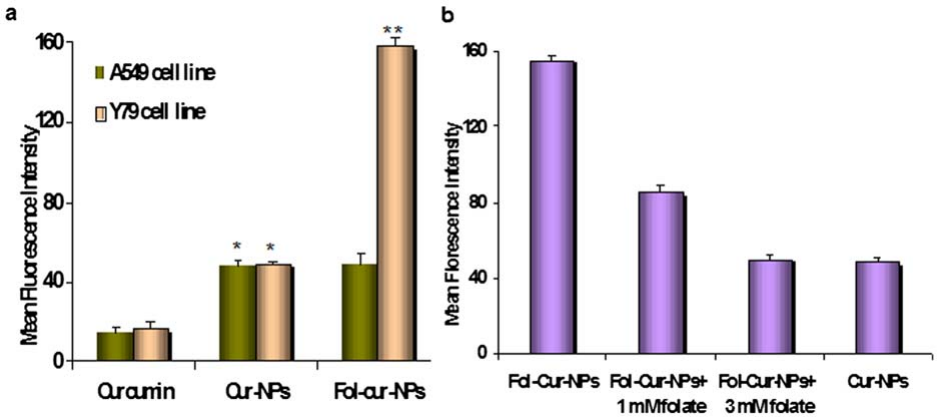
Uptake study by flow cytometry. Conjugation of folate on to nanoparticle increases its uptake in Y79 (a). A suspension of curcumin and Cur-NPs, Fol-Cur-NPs (10 µg/ml) was incubated with folate receptor expressing Y79 and non-expressing A549 cells (100,000 cells) for 2 hrs, and uptake of NPs by the cells were determined with flowcytometer by measuring the mean fluorescence intensity value. Competitive inhibition of uptake of Fol-Cur-NPs by addition of free folate (b). Y79 cells were treated with 0–30 mM of free folate for 1 h prior to incubation with curcumin and Cur-NPs, Fol-Cur-NPs and then subjected to above protocol. Mean fluorescence intensity of curcumin fluorescence in the cells was measured (n = 3; mean±s.e.m, (*) p<0.05 drug in solution versus drug loaded NPs, (**) p<0.005 drug in solution versus Fol-Cur-NPs).

**Figure 5:**
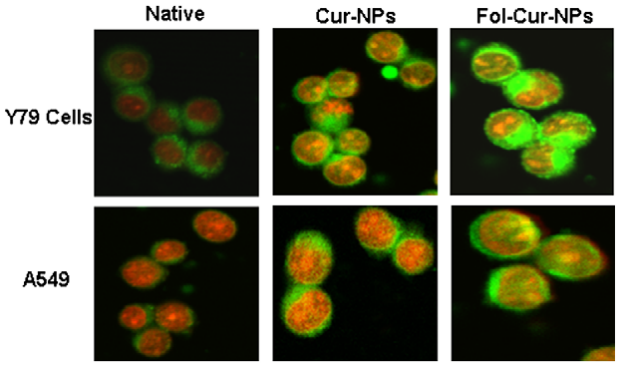
Qualitative uptake analysis by confocal microscopy. Cells were treated with native curcumin, Cur-NPs, Fol-Cur-NPs (10 µg/ml) and incubated for 2 hrs. Fol-Cur-NPs showed augmented fluorescence activity compared to native drug and unconjugated NPs in Y79 cell line, while no significant difference in uptake of Fol-Cur-NPs compared to Cur-NPs was evident in A549 cell line. Data represented as mean±s.e.m., n = 3.

Using the intrinsic fluorescence property of curcumin, a comparative analysis of cellular uptake behavior of Fol-Cur-NPs, Cur-NPs and native curcumin were performed in folate receptor expressing Y79 cells and folate receptor non-expressing A549 cells by flow cytometer and confocal laser scanning microscope. The relative extent of cellular uptake of native curcumin, Cur-NPs and Fol-Cur-NPs was presented in Figure 4a in terms of mean fluorescence intensity (MFI) exhibited by the cells. As evident from the figure, cellular uptake of Fol-Cur-NPs was nearly 3.3 times greater than unconjugated NPs and 9 times more than native curcumin in case of Y79 cell line. It is worth mentioning that, there was no substantial uptake of Fol-Cur-NPs in comparison to non-functionalized NPs in A549 cell line. The above results clearly indicate that, the superior uptake of Fol-Cur-NPs in comparison to native curcumin as well as Cur-NPs in Y79 cells is due to folate receptor mediated endocytosis. To verify that the uptake of Fol-Cur-NPs is mediated via folate receptor a competition of receptor mediated uptake experiment using different amounts of free folate in medium together with the Fol-Cur-NPs was performed. The specificity of folate receptor mediated internalization of folate conjugated NPs was evident from the decreased MFI value with increasing concentration of free folate (Figure 4b). The inhibition in uptake of Fol-Cur-NPs by free folate in a dose dependent manner demonstrates that the particle uptake is mediated via folate receptor in Y79 cell line. Figure 5, represents the qualitative uptake study of Fol-Cur-NPs, Cur-NPs and native curcumin in Y79 and A549 cell lines by confocal microscopy. Our result demonstrated that, nanoformulation showed augmented fluorescence activity, the activity being maximum with Fol-Cur-NPs in Y79 cell line at 2 hrs of incubation period. It is noteworthy to mention here that, A549 cells treated with Fol-Cur-NP and Cur-NPs showed similar fluorescence activity, while above nanoformulations exhibited augmented fluorescence activity compared to native curcumin. This visualization result was consistent with those presented in the flow cytometry analysis.

### Enhanced cytotoxicity of folate functionalized dual drug loaded NPs

**Figure 6:**
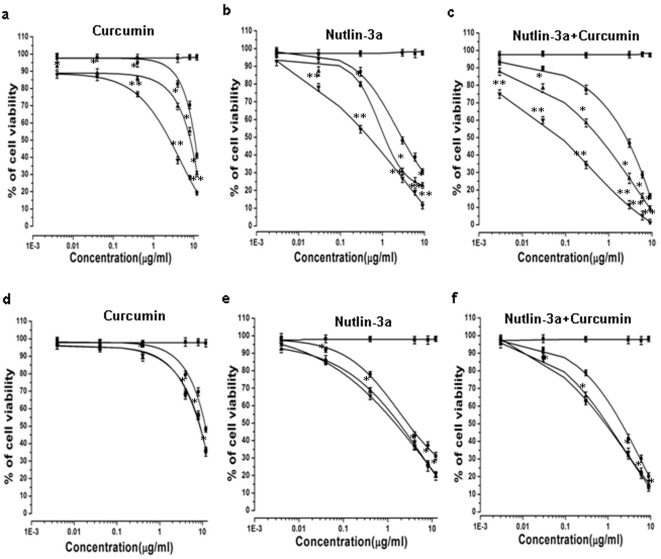
Cytotoxicity analyses. Dose dependent cytotoxicity study of curcumin or/and nutlin-3a in Y79 cells (a), (b), (c) and A549 cells (d), (e), (f). Different concentration of nutlin-3a or/and curcumin either as solution or encapsulated in NPs (NPs, Fol-NPs) and void NPs were added to wells with medium. The extent of growth inhibition was measured at 5^th^ day using MTT assay and inhibition was calculated with respect to untreated controls cells. Data are expressed as mean±s.e.m., n = 6, (*) p<0.05 drug in solution versus drug loaded NPs, (**) p<0.005 drug in solution versus Fol-NPs. ▪ Void NPs, • Native drug, ▴ Drug loaded NPs, ▾ Folate conjugated NPs.

Enhanced therapeutic potentiality of drug loaded polymeric NPs is the outcome of their cellular uptake and intracellular distribution [32]. In the present study, the chemosensitivity of the nanoformulation (unconjugated and conjugated NPs) as compared to native drugs was investigated at dose dependent manner for 5 days in folate receptor expressing Y79 cells and folate receptor negative A549 cells. It can be perceived from MTT assay that, Fol-Nut-NPs and Fol-Cur-NPs exhibited significantly higher cytotoxicity compared to native drugs or unconjugated drug loaded NPs at all drug concentration in Y79 cells (Figure 6a, b). The IC50 of Fol-Nut-NPs in Y79 cells was found to be 6 and 2.4 times lowered compared to native drug and unconjugated NPs respectively (Table 3). Whereas, exposure of cells with Fol-Cur-NPs lowered the IC50 value to 4.1, 3.2 times as compared to free curcumin and Cur-NPs respectively (Table 3). It is noteworthy that, cells treated with drugs in combination (nutlin-3a and curcumin either in solution or unconjugated/folate conjugated nanoformulations) showed augmented cytotoxicity, compared to single drug treatments (Figure 6c). The IC_50_ of Fol-Nut-Cur-NPs was found to be 35 and 8.6 times lower than native drugs in combination and uncojugated dual drug loaded NPs (Table 3). This clearly indicates that Fol-Nut-Cur-NPs elicited enhanced anti-proliferative activity as compared to dual drugs in solution or encapsulated in unconjugated NPs. To test the hypothesis that, enhanced cytotoxicity of folate conjugated NPs over unconjugated NPs is due to higher uptake of the conjugated system; we have studied its efficacy in a folate receptor negative cell line A549 and found that there is no significant difference in IC_50_ of Fol-NPs compared to its unconjugated counterpart (Figure 6d, e, f, Table 3). This clearly indicates that, the augmented cytotoxicity associated with folate conjugated NPs may be attributed to folate receptor mediated enhanced endocytosis.

**Table 3:** Cytotoxic effect of drug loaded nanoformulations.

Treatments	IC_50_ (µg/ml)	
	Y79 cells	A549 cells
Curcumin	10.70	11.73
Cur-NPs	8.26	8.64
Fol-Cur-NPs	2.56	8.80
Nutlin-3a	2.86	2.76
Nut-NPs	1.12	1.77
Fol-Nut-NPs	0.47	1.45
Nutlin+Curcumin	2.5	2.09
Nut-Cur-NPs	0.6	0.95
Fol-Nut-Cur-NPs	0.07	0.86

### Cell cycle analysis

**Figure 7:**
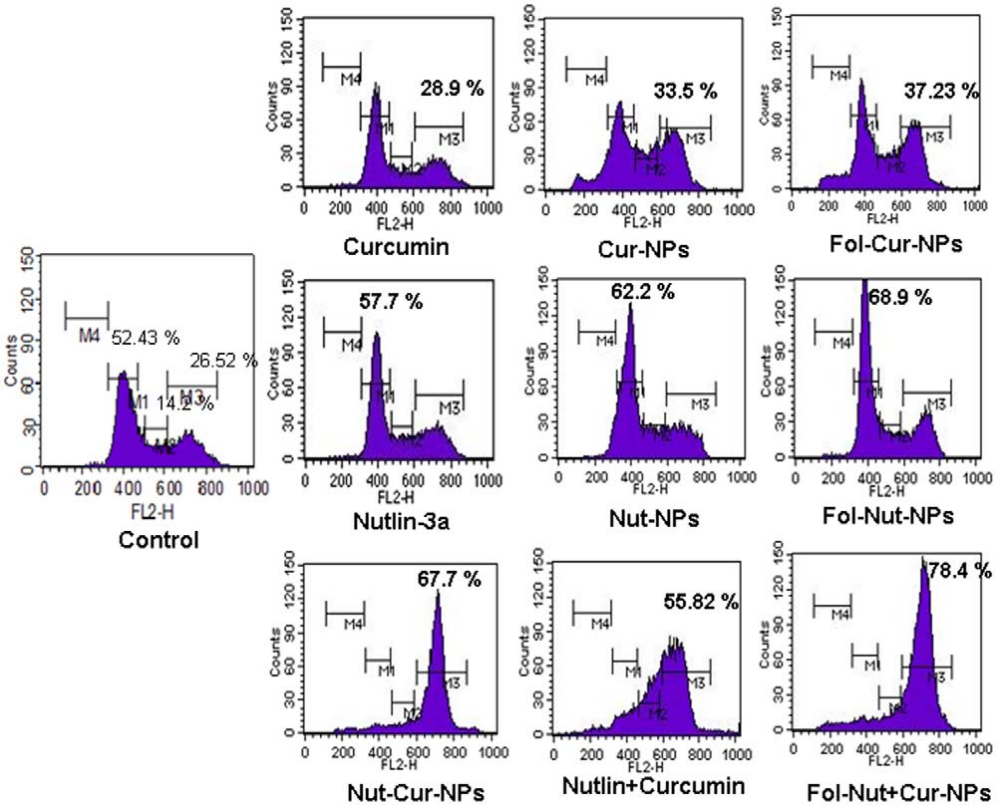
Effect of native drug and drug loaded NPs and folate conjugated NPs as single agents or in combination on cell cycle distribution of Y79 cells. 1×10^5^ cells/ml were treated with native nutlin-3a, Nut-NPs, Fol-Nut-NPs (1.5 µg/ml), curcumin, Cur-NPs, Fol-Cur-NPs (2 µg/ml) and Nutlin+Curcumin, Nut-Cur-NPs, Fol-Nut-Cur-NPs (1.5 nutlin+2 µg/ml curcumin). For cell cycle analysis, after 24 hrs 500 µl of hypotonic propidium iodide solution was added to treated cells, incubated at room temperature in dark for 1 h before flow cytometric analysis. Content of DNA is represented on the x-axis; number of cells counted is represented on the y-axis (n = 3). The region marked M1, M2, M3 and M4 represent G1, S, G2 and G_0_ phase respectively, of the cell cycle.

Both the chemotherapeutic agents (nutlin-3a and curcumin) are potent inducer of cell cycle arrest in G1 and/or G2 phase of cell cycle [28], [29], [36]. To determine whether co-administration of curcumin and nutlin-3a synergistically acts to enhance cell cycle arrest in Y79 cells, cell cycle analysis were performed following drug treatments for 24 hrs. From the result it becomes evident that, nutlin-3a (single formulations) cause G1 arrest while curcumin (single formulations) exhibited G2 phase arrest in Y79 cells (Figure 7). Notably, folate conjugated drug loaded NPs either Fol-Nut-NPs or Fol-Cur-NPs showed augmented activity than unconjugated counterpart. Interestingly, dual treatment of cells with both the drugs in solution or nanoformulations resulted in G2 phase arrest of cell cycle, the maximum arrest being exhibited by Fol-Nut-Cur-NPs.

### Drug-induced perturbation in mitochondrial transmembrane potential

**Figure 8:**
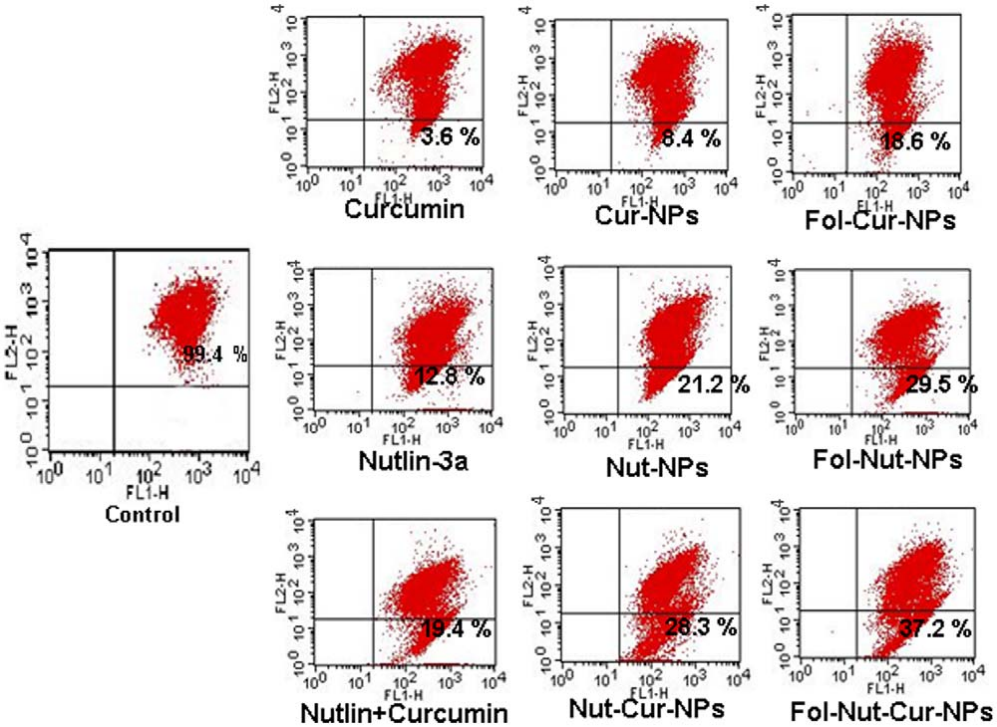
Detection of early induction of apoptosis was done using JC-1 dye by flow cytometry. Briefly, 1×10^5^ cells/ml were treated with native nutlin-3a, Nut-NPs, Fol-Nut-NPs (1.5 µg/ml), curcumin, Cur-NPs, Fol-Cur-NPs (2 µg/ml) and Nutlin+Curcumin, Nut-Cur-NPs, Fol-Nut-Cur-NPs (1.5 nutlin+2 curcumin µg/ml) for 48 hrs. Cells treated with only medium was used as controls. After completion of incubation period, cells were treated with JC1 staining solution (1 µg/ml in warm DPBS) and incubated at 37°C for 20 min in a CO2 incubator. After incubation period, cells were examined by flow cytometer. Data represented as mean±s.e.m., (n = 3). Folate targeted dual drug loaded NPs showed higher loss of MMP in Y79 cells compared native drugs (single or in combination) and unconjugated single or dual drug loaded NPs.

Loss of MMP can be detected by a fluorescent dye JC-1, which forms J-aggregates or monomers, depending on the state of mitochondrial membrane potential, with the emissions of the two dye forms detectable by flow cytometry at 585 nm (FL2) or 530 nm (FL1), respectively [37]. The high mitochondrial membrane potential of normal cells loaded with JC-1 allows for the formation of J-aggregates, as the mitochondrial membrane potential is loss, these aggregates dissipate into monomers. Figure 8 shows the loss in MMP of Y79 cells incubated with nutlin-3a or/and curcumin (in solution or nanoformulations), as the population of cells shifts to a lower JC-1 aggregate state and a concurrent higher JC-1 monomer state. As evident from the result, cells incubated with 1.5 µg/ml of Fol-Nut-NPs show loss of MMP in an enhanced way as compared to native drug or unconjugated counterpart. Similarly, with 2 µg/ml of curcumin treatment, Y79 cells treated with Fol-Cur-NPs demonstrated augmented loss of MMP as compared to same concentration of native curcumin and unconjugated NPs. It is noteworthy to mention that, drug in combination (as native form or nanoformulations) depicted an augmented loss in MMP compared to single drug treatments and Fol-Nut-Cur-NPs shows synergistic effect with enhanced therapeutic ability than native drug in combination or uncojugated counterpart.

### Supra-additive induction of apoptosis mediated by folate decorated dual drug loaded NPs

**Figure 9:**
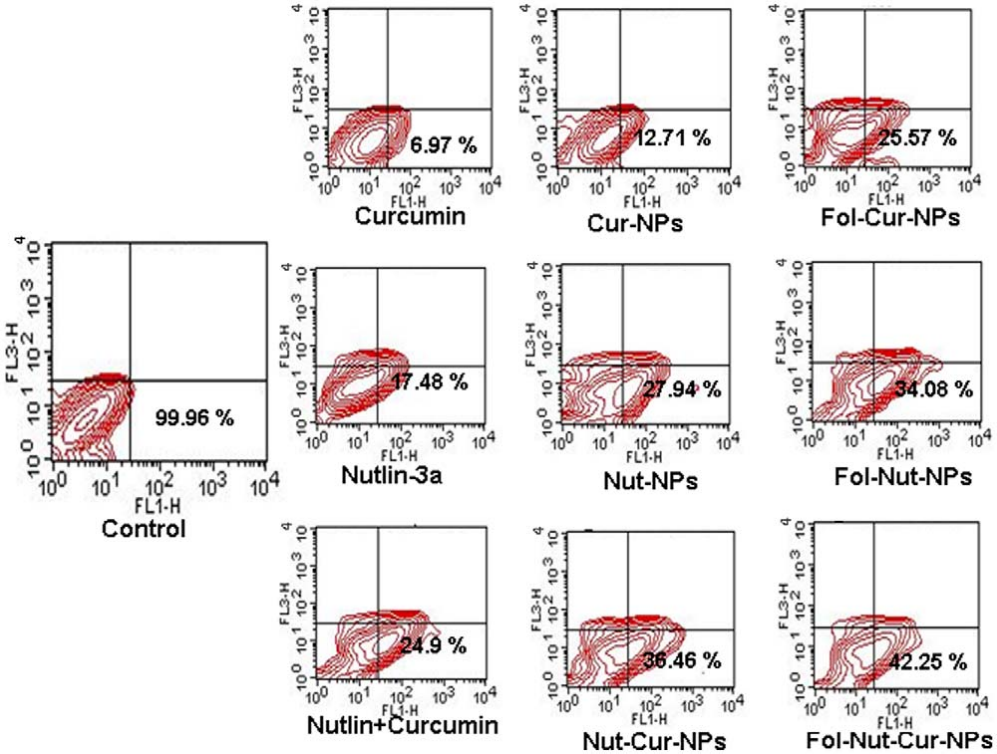
Induction of apoptosis in Y79 cell line nutlin-3a or/and curcumin (in solution or nanoformulation. 1×10^5^ cells/ml were incubated with native nutlin-3a, Nut-NPs, Fol-Nut-NPs (1.5 µg/ml), curcumin, Cur-NPs, Fol-Cur-NPs (2 µg/ml) and Nutlin+Curcumin, Nut-Cur-NPs, Fol-Nut-Cur-NPs (1.5 nutlin+2 curcumin µg/ml) for 48 hrs and apoptosis percentage was measured using standard Annexin V-FITC method. Data represented as mean±s.e.m., (n = 3).

In our studies, we found that cell cycle arrest and loss of MMP ultimately result in apoptosis of the cells (Figure 9). The result depicts the presence of a significant fraction of apoptotic cell population following treatment with various drugs individually (in solution or nanoformulation) or in combination (in solution or nanoformulation). Cells treated with 1.5 µg/ml Fol-Nut-NPs showed enhanced apoptotic activity compared to native drug. In a similar experiment, cells treated with Fol-Cur-NPs (2 µg/ml) elicited significant fraction of cell death following apoptosis than unconjugated substitute and native curcumin. Combination therapy act synergistically, as evident from the higher apoptotic cell death following simultaneous treatment with both the drugs (in solution or entrapped in NPs). It is worth mentioning that folate conjugated dual drug loaded NPs was competent enough to induce a more profound apoptotic cell death.

### Investigation of apoptosis inducing pathways following combination therapy

**Figure 10:**
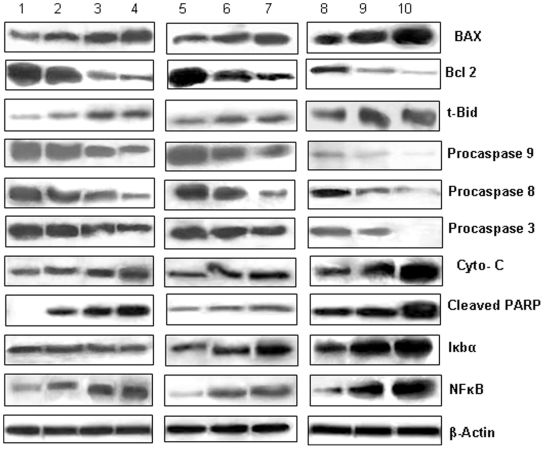
Study of apoptosis regulatory proteins by western blot analysis. Y79 cells were incubated with native nutlin-3a, Nut-NPs, Fol-Nut-NPs (1.5 µg/ml) or curcumin, Cur-NPs, Fol-Cur-NPs (2 µg/ml) or Nutlin+Curcumin, Nut-Cur-NPs, Fol-Nut-Cur-NPs (1.5 nutlin+2 curcumin µg/ml) for 48 hrs time period, and various apoptosis associated proteins were analyzed by western blotting. Lane 1, 2, 3, 4, 5, 6, 7, 8, 9, and 10 represents control, native nutlin-3a, Nut-NPs, Fol-Nut-NPs, curcumin, Cur-NPs, Fol-Cur-NPs, Nutlin+Curcumin, Nut-Cur-NPs, Fol-Nut-Cur-NPs respectively. Experiment was performed in triplicates.

Resistance to apoptosis is a hallmark of cancer, with both the loss of proapoptotic signals and the gain of anti-apoptotic mechanisms contributing to tumorigenesis. Most of the current chemotherapeutic agents exert their antitumor effect by triggering apoptosis signal transduction pathways (intrinsic and/or extrinsic) in cancer cells [38]. In the present study, we aim to define, which apoptotic pathway get activated in Y79 cells following treatment with drug in combination (in native form or nanoformulation) or single formulations. The result indicates the involvement of components of both the pathways to initiate apoptosis. Figure 10 shows down regulation of procaspases 8, 3 respectively and cleavage of PARP, which indicates towards the activation of extrinsic pathway. Significant down regulation of above proteins was evident in cells exposed to Fol-Nut-NPs or Fol-Cur-NPs compared to their unconjugated counter part and native drugs. It is worth mentioning that, drug in combination show better result and a more profound effect was exhibited by Fol-Nut-Cur-NPs. Mitochondria play an essential role in apoptosis triggered by various chemical agents [39]. Mitochondrial response during apoptosis includes down regulation of bcl2, activation of BAX and release of cytochrome c into the cytosol where it binds to Apaf-1, allowing the recruitment of caspase-9 and formation of an apoptosome complex, resulting in caspase-3 activation and execution of cell death by PARP cleavage [40]. To determine whether mitochontrial depolarization by nutlin-3a and/or curcumin leads to initiation of this cell death mechanism, we have investigated the key proteins associated with mitochondrial pathway of apoptosis by western blotting. Our result shows down regulation of bcl2 and over expression of anti-apoptotic proteins like tBid, BAX (that confer cytochrome c release by interacting with mitochondrial membrane) and indicates towards the involvement of mitochondrial pathway in apoptosis. The above finding was further supported by the presence of cytochrome c following drug treatments. Notably, down regulation of procaspases like caspase 9, caspase 3 followed by cleavage of PARP was also observed. It is noteworthy to mention that, enhanced expression of antiapoptotic proteins, cytoplasmic cytochrome c and significant reduction in expression of procaspases was evident in cells treated with Fol-Nut-Cur-NPs.

Many independent apoptosis regulatory pathways also operate in cells. One such pathway is the cell survival pathway regulated by NFκB and IκBα proteins. In the present study, we for the first time reporting that, both curcumin and nutlin-3a can effectively inhibit NFκB activity in Y79 cells. An increased expression of inactive NFκB was evident with Fol-Nut-Cur-NPs compared to respective unconjugated counterpart (single or dual formulations) (Figure 10). Interesting, expression of IκBα protein (associated with NFκB pathway) was found to be enhanced in cells treated with curcumin in solution or nanoformulation (Figure 10). However, no significant difference in its expression level was evident in cells exposed to nutlin-3a in single formulation. This clearly indicates that, nutlin-3a plays no role in regulating the other protein members associated with NFκB pathway. It is noteworthy to mention that; drug in combination shows up-regulation of IκBα in an enhanced manner, thus it point towards the fact that, our dual drug loaded nanoformulation is capable of activating multiple signaling pathways.

**Figure 11:**
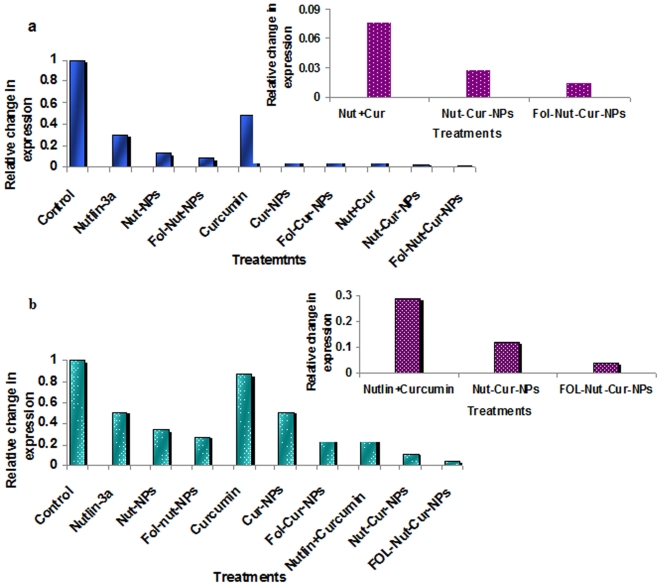
qRT PCR of bcl-2 and NFκB mRNA. Down regulation of bcl2 and NFκB gene expression by qRT PCR following treatment with native nutlin-3a, Nut-NPs, Fol-Nut-NPs (1.5 µg/ml), curcumin, Cur-NPs, Fol-Cur-NPs (2 µg/ml) and Nutlin+Curcumin, Nut-Cur-NPs, Fol-Nut-Cur-NPs (1.5 nutlin+2 curcumin µg/ml) for 48 hrs.

Induction of apoptosis by down-regulation of anti-apoptotic gene bcl 2 and NFκB bcl-2 is the prominent member of a family of proteins that are responsible for deregulation of apoptosis and prevention of death in cancer cells [41], [42]. bcl-2 overexpression and/or activation has been correlated with resistance to chemotherapy and radiotherapy [43]. Notably, overexpression of bcl-2 protein in retinoblastoma, confer drug resistance and make the disease more aggressive [3]. In the present study, we have shown the enhanced down regulation of bcl-2 protein expression in cells treated with drug in combination over their respective single drug formulation (Figure 10). Notably, Fol-Nut-Cur-NPs elicited more profound effect compared to unconjugated counterpart and native drug in combination. To confirm the above finding at transcription level we studied the expression of bcl-2 mRNA following different treatment by real time RT-PCR. Our result indicates an enhanced down regulation of mRNA expression in cells treated with Fol-Nut-Cur-NPs compared to unconjugated counterpart substitute and native drug (Figure 11a). Like bcl-2, cell survival protein NFκB plays an important role in signal transduction pathway regulating cell survival and apoptosis. In the present study, we are for the first time reporting that both curcumin and nutlin-3a was found to be effective in inhibiting NFκB transcriptional activity in Y79 cells. A decreased expression of NFκB mRNA was evident with Fol-Cur-NPs and Fol-Nut-NPs compared to respective unconjugated counterpart (Figure 11b). Interestingly, drug in combination was potent enough in inactivating NFκB than single drug treatments and Fol-Nut-Cur-NPs exhibited 7.2 and 2.8 fold lower expression of mRNA compared to unconjugated NPs or native drug in combination respectively. Our result disclose the fact that, at transcription level also, folate decorated dual drug loaded NPs was efficient to target many key pathways involved in regulation of apoptosis.

## Discussion

Drug resistance represents a major cause for therapeutic failure and death in retinoblastoma treatment. An important cause behind the execution of resistance by this overwhelming disease is the enhanced cellular efflux of a wide variety of structurally distinct classes of chemotherapeutic agents because of overexpression of the MDR proteins like MRP-1 and LRP etc. Addition to that, overexpression of antiapoptotic protein bcl-2, NFκB and inactivation of many signaling pathways like MDM2 mediated inactivation of p53 proteins, mutation of pRb gene notably contribute to drug resistance in retinoblastoma [3], [4]. Considering the relative importance of efflux transporters to execute drug resistance in diverse cancer, many research activities have been conducted till date to circumvent above lacunae by exploring the role of curcumin in modulating MDR activity. In three independent studies, Chearwae *et al.* have shown that, curcumin could effectively inhibit the function and expression of the major MDR proteins (P-gp, MRP-1, BCRP) in various drug resistance cell line *in vitro* [11]–[13]. Recently, Thiyagarajan *et al.* for the first time have investigated the role of curcumin in modulating LRP expression in Y79 cell line and found that under *in vitro* system, curcumin can efficiently down regulate LRP protein expression, thereby enhances the cytotoxicity of anticancer drug etoposite [7]. Although, curcumin has shown its potency as MDR modulator *in vitro*, however its clinical application is greatly limited by its poor aqueous solubility, poor bioavailability and short plasma half life etc. [17]. Thus, strategy to enhance the bioavailability and stability of curcumin is warranted to explore this molecule as MDR modulator clinically. Recently, our group has tried to increase the bioavailability of native curcumin to many folds by encapsulating it in nanoparticulate system in an animal model [19]. Therefore, we hypothesize that, by encapsulating curcumin in polymeric nanoparticulate system, we can augment the MDR modulatory activity of curcumin *in vitro*/*in vivo* and propose that such a therapeutic strategy can open new horizon for the treatment of cancer with drug resistance phenomena in clinical setup. A strategy that has been investigated in recent year to enhance the therapeutic potentiality of drug loaded carrier system is co-delivery of two potential therapeutic agents which act either additively or synergistically to kill tumor cells in an enhanced way. In the present state of affairs, we take advantage of combination therapy and aimed to develop a therapeutic nanocarrier capable of drug delivery and molecular targeting for simultaneous reversion of drug resistance by functional inhibition of MDR proteins and hitting of multiple signaling targets associated with cell survival or apoptosis. With this concept in mind, we developed a folate targeted polymeric nanopartices co-loaded with anticancer drug nutlin-3a (a potent activator of p53, also inactivate NFκB) and chemo-sensitizer curcumin (effectively modulate MRP-1 and LRP activity) to act additively or synergistically for enhanced therapeutic efficacy in multidrug resistance Y79 cells. We further, highlighted the role of curcumin in modulating the functionality of LRP and MRP1 in Y79 cell line.

For developing an efficient drug delivery vehicle, physicochemical characterization of the drug loaded nanosystem is of prime importance, as it significantly influences the *in vitro* and *in vivo* performance of the entrapped drug. In the present work, we have developed small sized NPs, which could show higher accumulation at tumor sites due to their escape by the reticuloendothelial system [44]. A carrier with sustained release property at the site of action would enhance the therapeutic efficiency of the anticancer drug as well as could sustain its therapeutic effect. Our nanoparticulate formulation solely exhibit a sustain release phenomena, under *in vitro* condition as depicted in Figure 1c and d. Rapid initial release is attributed to the drug which is adsorbed or weakly bound to large surface area of the nanoparticles, and the slow release could be caused by diffusion of the drug inside the nanoparticles [45].

As our aim is to reverse MDR in retinoblastoma cell line Y79 to enhance the therapeutic potentiality of nutlin-3a, we assessed the effect of curcumin on expression of MRP-1 and LRP mRNA (Figure 3a, b,). Our result demonstrated a dose dependent inhibition of MRP-1 and LRP gene expression following curcumin treatment. The MDR gene suppressing activity of curcumin was further assessed at protein level by western blotting and the result clearly indicates down regulation of MRP-1 and LRP protein with increasing curcumin concentration (Figure 3c). The protein expression study was in accordance with gene expression analysis and point towards the fact that curcumin is a potent modulator of MDR proteins. Similar type of work has been conducted by many research groups where they have found that; curcumin is effective enough to modulate function and expression of various MDR proteins (Pgp, MRP-1, LRP etc.) in a dose or time dependent manner [7], [12], [46]. Noteworthy, it has also been reported that curcumin get metabolized at longer time points and the active metabolite like tetrahydrocurcumin plays the key role in modulation of MDR protein and gene expression [47]. To this end, curcumin or its active metabolites, that actually take part in regulating MDR exhibited by Y79 cell needs further investigation.

**Figure 12:**
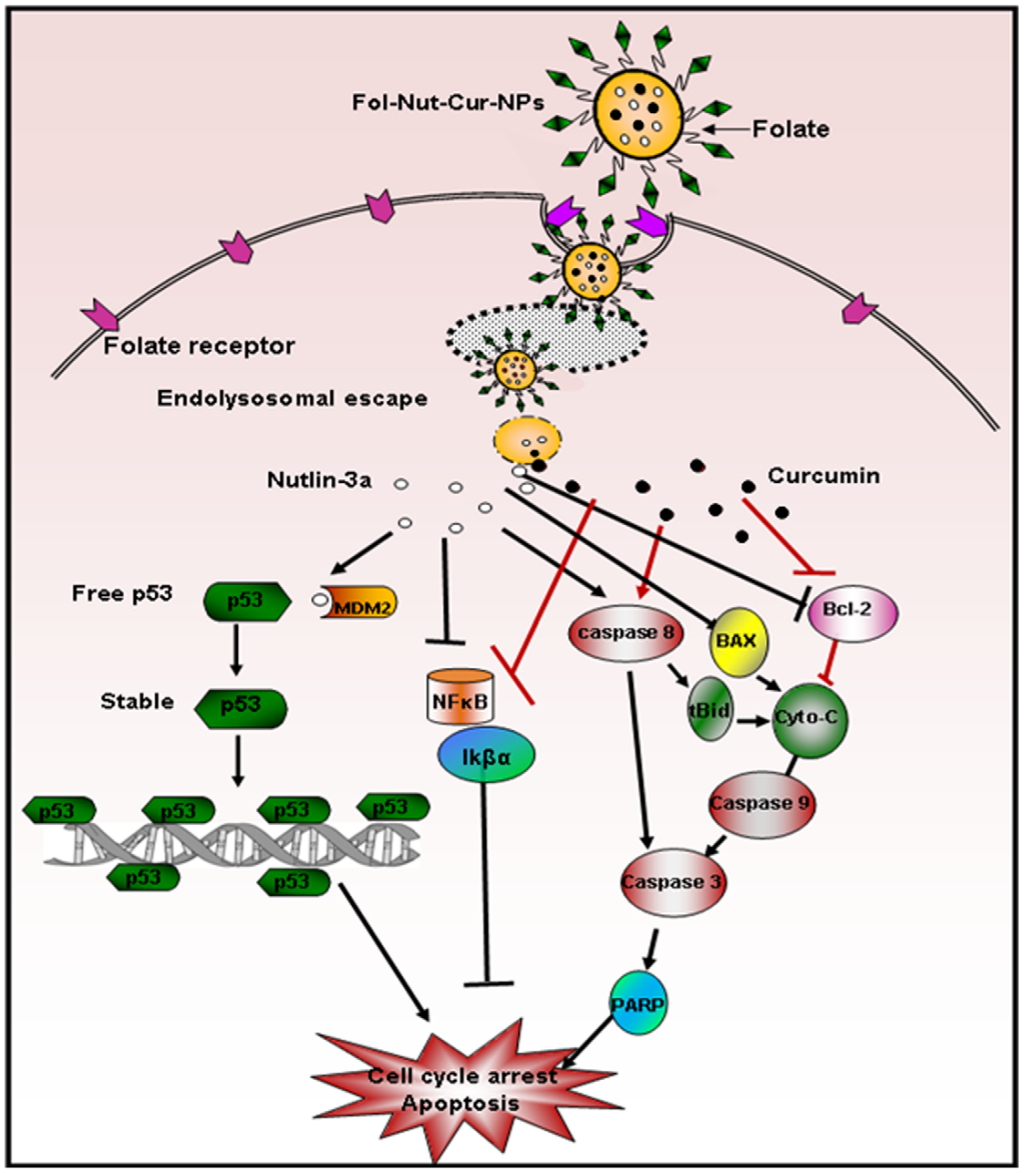
Schematic presentation of proposed signaling pathway activated by folate targeted dual drug loaded nanoparticles. Folate conjugated NPs loaded with nutlin-3a (o) and curcumin (•) synergistically activates major apototic proteins or down regulates major antiapoptotic proteins involved in apoptosis pathway.

The enhanced chemotherapeutic activity of the ligand targeted carrier system was assessed in terms of cellular uptake efficiency of the carrier system. Our results clearly demonstrate that, uptake of Fol-Cur-NPs was significantly greater than that of the unconjugated particle and native drug in folate receptor expressing Y79 cells (Figure 4a). Further, the specificity of folate receptor mediated internalization of conjugated NPs was evident from the decreased uptake of Fol-Cur-NPs with increasing concentration of free folate (Figure 4b). The greater uptake of Fol-Cur-NPs in Y79 cells clearly indicates the role of higher folate receptor expression and receptor mediated binding and uptake of conjugated NPs. Intensive investigation have been done on folate-receptor mediated uptake of drugs and macromolecules in recent years. Elegant work conducted by Wang *et al.* have demonstrated increased level of uptake of folate conjugated paclitaxel loaded micelles compared to unconjugated micelles in MCF7/ADR cells, thus proving that targeted delivery of carrier system results in enhanced and discriminatory drug uptake by the cancer cells [48]. Besides this, the enhanced uptake level of folate conjugated NPs may be due to the reduced exocytosis of conjugated system than unconjugated NPs [49]. Enhanced intracellular uptake of Fol-Cur-NPs by Y79 cells was substantially confirmed by confocal microscopy, where augmented fluorescence activity of cells exposed to Fol-Cur-NPs indicates that conjugated system were internalized more in comparison to native drug and unconjugated Cur-NPs (Figure 5). Simultaneous delivery of anticancer drug and chemosensitizer ultimately aims towards the better efficacy of drug in terms of cellular cytotoxicity. By encapsulating anticancer drug nutlin-3a and chemosensitizer like curcumin with inherent cytotoxic activity, we hypothesized to achieve enhanced antiproliferative activity in drug resistance Y79 cells. Our result demonstrated a sharp discrimination in cell inhibition with dual drug loaded nanoformulations or native drug in combination compared to respective single drug counter parts, thus stressing the role of curcumin in combination in inhibiting MDR activity (Figure 6a, b, c). Similar type of result was obtained by Patil *et al.* when they used the cytotoxic drug paclitaxel and chemosensitizer tariquidar [21]. Further, the enhanced cytotoxicity exhibited by folate targeted dual drug loaded NPs as compared to unconjugated dual drug loaded NPs or native drug in combination could be attributed to enhanced accumulation of folate decorated drug loaded NPs. The above hypothesis was supported by the uptake result in which enhanced accumulation of Fol-Cur-NPs compared to Cur-NPs was evident in folate receptor expressing Y79 cells, while no remarkable difference in cellular accumulation of Fol-Cur-NPs and Cur-NPs was found in folate receptor non-expressing A549 cells (Figure 4 and 5). The concept of receptor mediated enhanced accumulation leading to higher cytotoxicity was further confirmed by MTT assay of folate targeted dual drug loaded NPs and unconjugated or native counterpart in folate receptor nonexpressing A549 cell line (Figure 6d, e, f). Nutlin-3a and curcumin inhibits cell growth and proliferation primarily through cell cycle arrest and apoptosis inducing mechanism [36], [50]. In our studies, treatment of Y79 cells with Fol-Nut-NPs and Nut-NPs resulted in greater proportion of cell in G1 phase as compared to cells treated with drug in solution (Figure 7). Similarly, cells incubated with Fol-Cur-NPs, Cur-NPs showed higher proportion of cell in G2 phase compared curcumin in solution. Studies conducted by Park *et al.* suggested that NPs are more effective in controlling cell cycle analysis than native drug in A549 and HeLa cells [51]. However, greater efficiency of folate conjugated drug loaded NPs in arresting more number of cells can be explained on the basis of increased intracellular drug level at the site of action for a longer period of time than native drug and unconjugated NPs [49], [52]. Interestingly, drug in combination showed G2 phase arrest (Figure 7). This issue can be further addressed by highlighting the role of p53 in cell cycle regulation. It is a well-known fact that p53-dependent cell cycle arrest at G1 phase is in part a consequence of up-regulation of p21 protein. p21 inhibits G1 cyclin dependent kinase, leading to a G0-G1 arrest of the cell cycle [53]. Further, p53 has been shown to induce G2 arrest by primarily perturbing the function of cyclin B1/cdc2 complex [54]. Additionally, activation of p53 protein also leads to expression of protein like 14-3-3σ involved in G2 phase arrest, thus ultimately results in arrest of cells at G2 phase [55]. As nutlin-3a is a potent activator of p53 protein it is anticipated that, enhanced accumulation of nutlin-3a and curcumin due to MDR reversing activity of curcumin may leads to enhanced cell arrest at G2 phase of cell cycle. Recent findings have suggested that alterations of mitochondrial function may be important for early indication of apoptosis [56]. In the present study, the augmented loss of MMP in Y79 cells following Fol-Nut-Cur-NPs treatment compared to unconjugated counterpart (drug in single or combination) or drug in solution (single or combination) can be attributed to increased drug accumulation due to receptor mediated endocytosis and reduced efflux because of MDR modulatory effect of curcumin (Figure 8). The molecular mechanism of nutlin-3a or curcumin mediated loss of MMP could be well explored by highlighting the role of cytochrome c (Cyt-c). Cyt-c is normally localized in the space between the outer and inner mitochondrial membrane and is only released into the cytosol in the setting of apoptosis [57]. The expression of cytoplasmic Cyt-c in Y79 cells as evident from our western result (Figure 10) supports the point that nutlin-3a or/and curcumin mediated release of Cyt-c which results in loss of MMP. From Figure 10, it can also be said that at molecular level greater accumulation of folate targeted dual drug loaded nanoformulation with MDR modulating activity results in higher expression of Cyt-c, resulting in enhanced loss of MMP.

Apoptosis is a key regulator of physiological growth control and regulation of tissue homeostasis but it is highly deregulated in cancer. Execution of apoptosis is regulated by plethora of cell signaling pathways and current chemotherapeutic agents solely exhibit their action by targeting these signaling pathways. Our results demonstrated higher fraction of apoptotic cell death in Y79 cells treated with Fol-Nut-NPs or Fol-Cur-NPs than unconjugated counterpart or native drug in solution following site specific sustained release pattern (Figure 9). It is noteworthy mentioning that, drugs in combination (either in solution or nanoformulation) elicited enhanced apoptotic activity compared to single drug treatment, the activity being maximum with Fol-Nut-Cur-NPs. The enhanced therapeutic ability of dual drug formulation over single drug can be addressed by the fact that, two or more drug in combination with distinct cellular targets are able to hit multiple signaling cascades simultaneously, thus ultimately causing enhanced apoptosis. In the present work, we anticipate that, our dual drug loaded nanoparticulate system also target multiple signaling pathways simultaneously and able to cause enhance apoptosis (Figure 12). The concept that, drug in combination hits multiple cellular targets and cause increased cell death was further confirmed by exploring the expression of various apoptotic or anti-apoptotic proteins by western blot analysis. In general two apoptotic signaling pathways (extrinsic and intrinsic) operates in cell to regulate the apoptosis mechanism [38]. The extrinsic mechanism of apoptosis involves activation of caspases like caspase 7, 8, 3 and finally cleavage of PARP. In a similar way intrinsic mechanism of apoptosis is triggered by activation of BAX, PUMA, NOXA followed by release of cytochrome c, activation of caspase 9, caspase 3 and PARP cleavage [38]. An interconnected mechanism of apoptosis that initiated as external pathway but end as internal mitochondrial pathways was mediated by cleavage of bid [38]. All these signaling pathways either act alone or merge to elicit apoptotic response. To investigate by which pathways our dual drug loaded NPs exhibit its apoptotic response, we performed western blot analysis. In the present work we found the expression of BAX and cytochrome c, down regulation in expression of procaspases 9, procaspase 7, procaspase 3 and expression of cleaved PARP in cells treated with all formulation either used as single agent or in combination. In addition to that expression of t-bid (the connector between extrinsic and intrinsic pathways) clearly indicates the combination effort of both the pathways in eliciting apoptotic response (Figure 10). Notably, folate functionalized nanosytems showed greater efficacy following site specific drug delivery, however enhanced activity of drug in combination in triggering apoptosis response could be attributed to the synergistic action of both the drugs.

In addition to LRP and MRP-1 in eliciting drug resistance in retinoblastoma, antiapoptotic protein bcl2 and cell survival protein NFκB also play significant role in multidrug resistance of retinoblastoma. Our result demonstrates significant down regulation of both the proteins following treatment with native drugs or nanoformulation (used as single agent or in combination (Figure 10). Notably, folate targeted dual drug loaded nanoformulation exhibited maximum inhibition due to enhanced uptake by receptor mediated endocytosis and reduced efflux of drug by modulation of MRP proteins. At the transcriptional level also expression of both the gene was down regulated by all the formulation, the suppression being maximum with Fol-Nut-Cur-NPs (Figure 11).

An ultimate goal in cancer therapy is to devise individually tailored treatment strategy that targets growth-promoting pathways and circumvents drug resistance. With this aim, in the present study we formulated dual drug loaded nanocarrier with property of reversing drug resistance and ability to targets many growth-promoting pathways. Further, for site specific delivery of therapeutic payload we functionalized the carrier with folate ligand. Our results clearly indicate that, nutlin-3a along with curcumin in PLGA nanoparticles resulted in significantly enhanced cellular accumulation of drugs, and greater cytotoxicity in MDR over expressing cells. Co-delivery of both therapeutic agents synergistically acts to induce better cell cycle arrest, loss of MMP and enhanced apoptosis by triggering many signaling pathways. Overall, we anticipate that this drug combination will show new horizon in treatment of cancer that exhibits multidrug resistance phenomenon.

## Supporting Information

Methods S1 **Fourier transform infrared (FTIR) spectral analysis of folic acid, void PLGA NPs, Fol-PLGA-NPs.**
(DOCX)

Results S1 **FTIR spectra of free folic acid, void PLGA-NPs and Fol-PLGA-NPs.** Western blot analysis on effect of curcumin loaded nanoformulation in comparison to native curcumin on the expression of LRP and MRP-1 proteins.
(DOCX)

Figure S1 **FTIR spectroscopy of free folic acid (I), Void PLGA-NPs (II) and Fol-PLGA-NPs (III).**
(TIF)

Figure S2 **Expression study of LRP and MRP-1 protein by western blotting.** Y79 cells were treated with 2 µg/ml of native curcumin or equivalent amount of curcumin entrapped in nanoformulation for 48 hrs and protein expression was investigated by western blotting. 1: control, 2: Native Curcumin, 3: Cur-NPs, 4: Fol-Cur-NPs.
(TIF)
