# Supplementary material for: Folate Decorated Dual Drug Loaded Nanoparticle: Role of Curcumin in Enhancing Therapeutic Potential of Nutlin-3a by Reversing Multidrug Resistance
Source: PLoS One. 2012 Mar 21;7(3):e32920. doi: 10.1371/journal.pone.0032920 (PMC3310050; doi:10.1371/journal.pone.0032920)
Supplement: Methods S1 — Fourier transform infrared (FTIR) spectral analysis of folic acid, void PLGA NPs, Fol-PLGA-NPs. (DOCX) [file pone.0032920.s001.docx]

**Method**

***FTIR analysis***

The Fourier transform infrared (FTIR) spectra for folic acid, void PLGA NPs, Fol-PLGA-NPs were obtained from FTIR spectrophotometer SPECTRUM RX I (Model Spectrum 1, Perkin-Elmer, San Jose, California) for studying the surface modification of PLGA-NPs after folate conjugation to PLGA NPs surface. Briefly, the samples were pressed with potassium bromide to make a pellet by applying a pressure of 300 kg/cm2 before obtaining their IR absorption spectra. The spectra were detected in KBr disks over a range of 4000-500 cm^-1^.
