# Supplementary material for: Folate Decorated Dual Drug Loaded Nanoparticle: Role of Curcumin in Enhancing Therapeutic Potential of Nutlin-3a by Reversing Multidrug Resistance
Source: PLoS One. 2012 Mar 21;7(3):e32920. doi: 10.1371/journal.pone.0032920 (PMC3310050; doi:10.1371/journal.pone.0032920)
Supplement: Results S1 — FTIR spectra of free folic acid, void PLGA-NPs and Fol-PLGA-NPs. Western blot analysis on effect of curcumin loaded nanoformulation in comparison to native curcumin on the expression of LRP and MRP-1 proteins. (DOCX) [file pone.0032920.s002.docx]

**Results**

***FTIR analysis***

The FTIR analysis was performed to study the chemical modifications or changes that occurred in the polymer in the form of band stretching or bending due to conjugation of folic acid onto the NPs surface [1-3]. Figure S1, shows the FTIR spectra of free folic acid (I), void PLGA-NPs (II) and Fol-PLGA-NPs (III) respectively. The IR spectrum of Fol-PLGA-NPs in Figure S1 (III), shows peaks at 1426.05 cm^-1^, 512.47 cm^-1^ and 453.3 cm^-1^ similar to the peaks observed in folic acid (Figure S1, I). In addition, the peaks appearing at 1630.11 cm^-1^ of Figure S1, (III) corresponded to C=O amide bond , confirming the successful conjugation of folate to PLGA NP surface for improved targeting.

***Western blotting***

Effect of curcumin loaded nanoformulation on the expression of LRP and MRP-1 proteins in comparison to native curcumin was investigated by western blot analysis (Figure S2). The result clearly indicate a significant inhibition in expression of above proteins in an enhanced manner following treatment with 2 μg/ml of Fol-Cur-NPs compared to native drug and unconjugated counterpart. This finding suggests the applicability of nanoformulation in modulating MDR protein expression for overcoming multidrug resistance.

***References***

1. Arya G, Vandana M, Acharya S, Sahoo SK. Enhanced antiproliferative activity of Herceptin (HER2)-conjugated gemcitabine-loaded chitosan nanoparticle in pancreatic cancer therapy. Nanomedicine 2011;7:859-70.

2. Misra R, Acharya S, Dilnawaz F, Sahoo SK. Sustained antibacterial activity of doxycycline-loaded poly(D,L-lactide-co-glycolide) and poly(epsilon-caprolactone) nanoparticles. Nanomedicine (Lond) 2009;4:519-30.

3. Vandana M, Sahoo SK. Optimization of physicochemical parameters influencing the fabrication of protein-loaded chitosan nanoparticles. Nanomedicine (Lond) 2009;4:773-85.
