# Supplementary figures and images for: Folate Decorated Dual Drug Loaded Nanoparticle: Role of Curcumin in Enhancing Therapeutic Potential of Nutlin-3a by Reversing Multidrug Resistance
Source: PLoS One. 2012 Mar 21;7(3):e32920. doi: 10.1371/journal.pone.0032920 (PMC3310050; doi:10.1371/journal.pone.0032920)

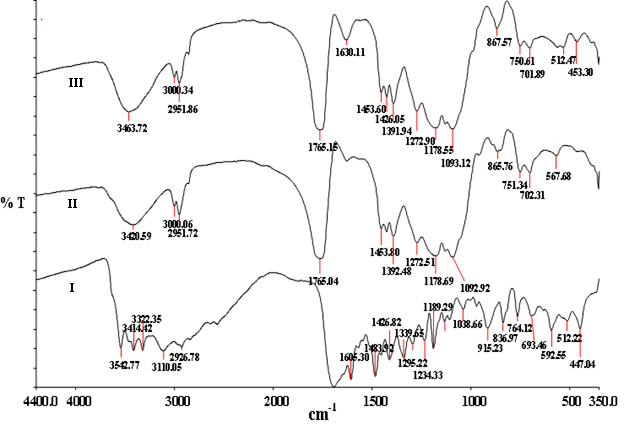

Supplement: Figure S1 — FTIR spectroscopy of free folic acid (I), Void PLGA-NPs (II) and Fol-PLGA-NPs (III). (TIF) [file pone.0032920.s003.tif]

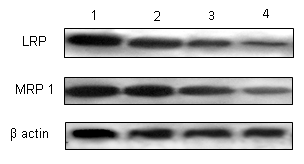

Supplement: Figure S2 — Expression study of LRP and MRP-1 protein by western blotting. Y79 cells were treated with 2 µg/ml of native curcumin or equivalent amount of curcumin entrapped in nanoformulation for 48 hrs and protein expression was investigated by western blotting. 1: control, 2: Native Curcumin, 3: Cur-NPs, 4: Fol-Cur-NPs. (TIF) [file pone.0032920.s004.tif]
